# Dosing and formulation of antenatal corticosteroids for fetal lung maturation and gene expression in rhesus macaques

**DOI:** 10.1038/s41598-019-45171-6

**Published:** 2019-06-21

**Authors:** Augusto F. Schmidt, Paranthaman S. Kannan, James P. Bridges, Alyssa Filuta, Dakota Lipps, Matthew Kemp, Lisa A. Miller, Suhas G. Kallapur, Yan Xu, Jeffrey A. Whitsett, Alan H. Jobe

**Affiliations:** 1 Division of Neonatology, Perinatal and Pulmonary Biology, Cincinnati Children’s Hospital Medical Center, Cincinnati, OH USA; 2 Department of Pediatrics, College of Medicine, University of Cincinnati, Cincinnati, OH USA; 3 School of Women’s and Infants’ Health, University of Western Australia, Perth, Australia; 4 Department of Anatomy, Physiology & Cell Biology, School of Veterinary Medicine, University of California, Davis, CA USA; 5 Division of Neonatology and Developmental Biology, David Geffen School of Medicine, University of California, Los Angeles, CA USA; 6 Division of Neonatology, Department of Pediatrics, University of Miami Miller School of Medicine, Miami, FL USA

**Keywords:** Preclinical research, Translational research, Paediatric research

## Abstract

Antenatal corticosteroids (ANS) are the major intervention to decrease respiratory distress syndrome and mortality from premature birth and are standard of care. The use of ANS is expanding to include new indications and gestational ages, although the recommended dosing was never optimized. The most widely used treatment is two intramuscular doses of a 1:1 mixture of betamethasone-phosphate (Beta-P) and betamethasone-acetate (Beta-Ac) – the clinical drug. We tested in a primate model the efficacy of the slow release Beta-Ac alone for enhancing fetal lung maturation and to reduce fetal corticosteroid exposure and potential toxic effects. Pregnant rhesus macaques at 127 days of gestation (80% of term) were treated with either the clinical drug (0.25 mg/kg) or Beta-Ac (0.125 mg/kg). Beta-Ac alone increased lung compliance and surfactant concentration in the fetal lung equivalently to the clinical drug. By transcriptome analyses the early suppression of genes associated with immune responses and developmental pathways were less affected by Beta-Ac than the clinical drug. Promoter and regulatory analysis prediction identified differentially expressed genes targeted by the glucocorticoid receptor in the lung. At 5 days the clinical drug suppressed genes associated with neuronal development and differentiation in the fetal hippocampus compared to control, while low dose Beta-Ac alone did not. A low dose ANS treatment with Beta-Ac should be assessed for efficacy in human trials.

## Introduction

Antenatal corticosteroids (ANS) are the major perinatal intervention to reduce the incidence of respiratory distress syndrome and neonatal mortality associated with preterm birth^1^. While ANS are considered to be safe, expanding treatments to gestations beyond 24 to 34 weeks may change the risk to benefit ratio^2^. A recent large trial demonstrated that ANS decreased the respiratory complications and the need for respiratory support for infants between 34 and 37 weeks gestational age^3^. ANS also decreased neonatal respiratory morbidity for elective C-section deliveries at term gestation^4,5^. ANS also are being used for periviable deliveries at 22–24 weeks gestation^6^. If widely adopted for these expanded indications and depending on the elective C-section rate, the majority of pregnancies worldwide could be treated with ANS^7,8^. However, the limited knowledge regarding the optimal formulation, dosing and potential delayed effects into adult life on outcomes such as metabolic syndrome suggests caution for the expanded use of ANS.

There also may be unrecognized adverse effects of ANS on different patient populations. The largest burden of prematurity is in low and middle-resource countries and in a recent large cluster-randomized trial ANS increased mortality for exposed infants with birthweight above the 5^th^ percentile^9,10^. Hypoglycemia also was increased with ANS exposure in late preterm newborns (with gestational ages between 34^0^ and 36^6^ weeks)^3^. In experimental studies ANS caused hippocampal degeneration and HPA axis dysfunction in macaques^11–13^ and decreased fetal and brain growth in sheep and rats^14–16^. These findings were consistent with observational studies showing that ANS impaired fetal growth^17^ and decreased neuronal density in the hippocampus of newborns^18^.

As with any medication, treatment strategies should maximize the benefits and minimize potential toxic effects. ANS are recommended by the World Health Organization to be given to the mother as intramuscular (IM) dexamethasone-phosphate (Dex-P), or a combination of equal parts of Beta-P and Beta-Ac (clinical drug)^19^. Phosphate and acetate formulations have distinct pharmacokinetic profiles and effects^20,21^. Phosphate preparations are rapidly dephosphorylated resulting in early high plasma concentrations and short half-lives in the mother and fetus. Beta-Ac is slowly deacetylated resulting in low peak plasma levels and long half-life^20^. In fetal sheep the clinical drug was superior to Dex-P or Beta-P alone for causing physiologic and biochemical maturation of the lung^22^. In contrast, a single weight-based dose of Beta-Ac improved maturation comparably to the clinical drug, with a decreased fetal exposure to Beta^23^.

Any pharmacological intervention for treating a pregnant woman to benefit the fetus should be considered high risk. To translate a new ANS therapy from animal models to humans, a validation in primates is desirable. We evaluated a lower dosing strategy using Beta-Ac for fetal lung maturation and transcriptional effects on the fetal lung and brain in the Rhesus macaque.

## Results

### Beta-Ac enhances fetal lung maturation at 5 days

**Figure 1:**
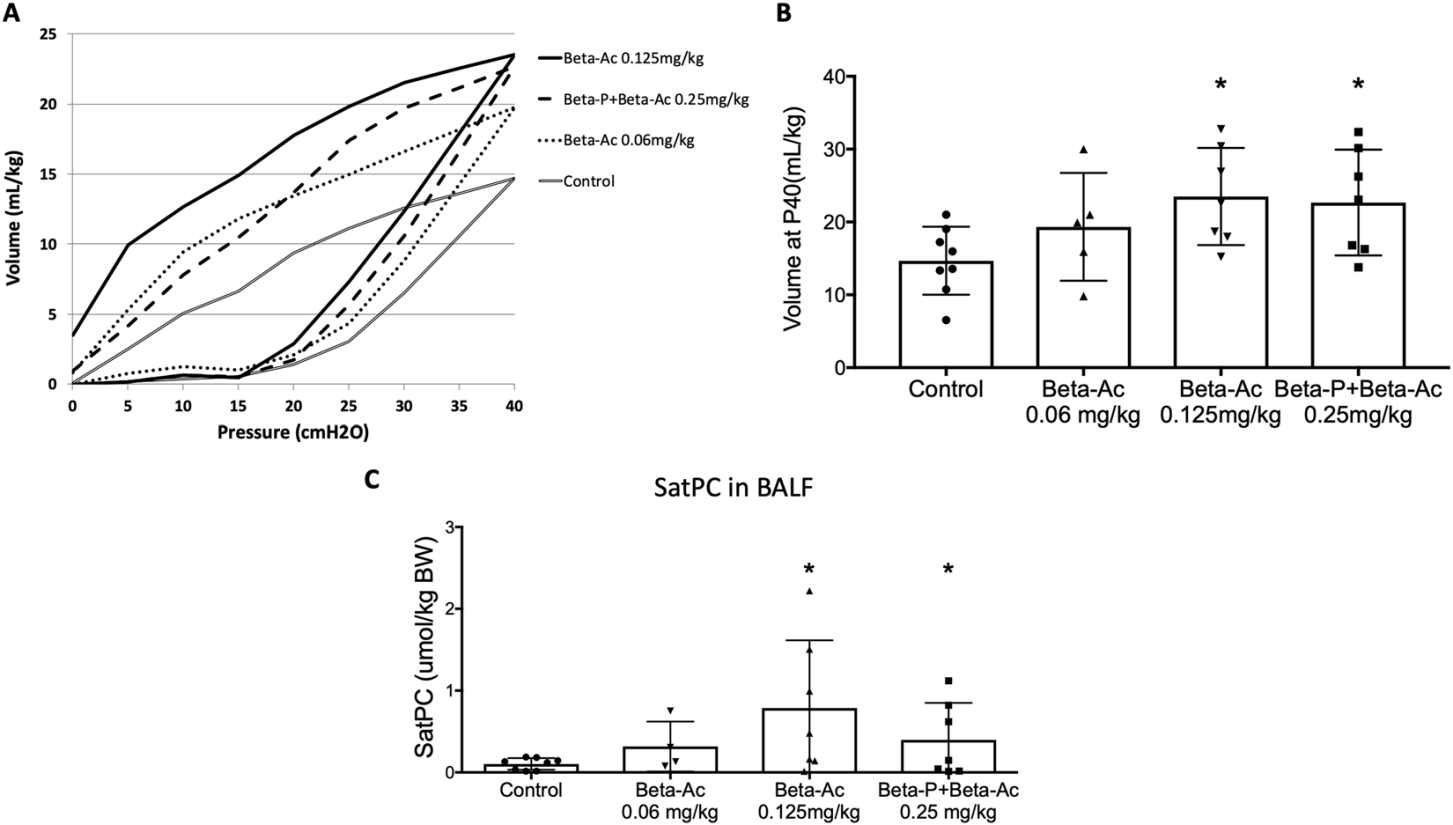
(**A**) Pressure-volume curves showing comparable improved static compliance after treatment with Beta-Ac (0.125 mg/kg) and the clinical drug. (**B**) Lung gas volumes were increased significantly for Beta-Ac 0.125 mg/kg and the clinical drug. (**C**) Saturated phosphatidylcholine (SatPC) concentration in the bronchoalveolar lavage fluid (BALF) increased with Beta-Ac 0.125 mg/kg and the clinical drug compared to control. *p-value < 0.05 vs. control.

To compare Beta-Ac with the clinical drug for fetal lung maturation, pregnant Rhesus macaques were treated with a single IM injection of either Beta-Ac (0.125 mg/kg or 0.06 mg/kg), saline (control) or the clinical drug (0.25 mg/kg) and delivered 5 days after treatment (Table 1). ACS induce fetal lung maturation by a combination of increased surfactant production, structural changes, and improved water clearance resulting in increased lung compliance. Both the clinical drug and Beta-Ac (0.125 mg/kg) improved static lung compliance (Fig. 1A,B). Beta-Ac 0.06 mg/kg inconsistently increased lung compliance with 3 out of 5 treated animals having pressure-volume curves similar to control. The main lipid component of surfactant is saturated phosphatidylcholine (SatPC), which can be used as a marker of lung surfactant content and indicator of biochemical lung maturation^24^. Both Beta-Ac (0.125 mg/kg) and the clinical drug increased the SatPC in the bronchoalveolar lavage fluid compared to control, while the increase with Beta-Ac (0.06 mg/kg) was not significant (Fig. 1C).

**Table 1:** Weight, gestational age, sex and cortisol of the animals. The groups were comparable. Cord plasma cortisol concentration measured for control animals and 5 days.

n	Control	Clinical drug		Beta-Ac		
		0.25 mg/kg		0.06 mg/kg	0.125 mg/kg	
		4 hours	5 days	5 days	6 hours	5 days
	8	3	7	5	3	7
Birth weight (g)	330 ± 35	317 ± 22	338 ± 39	302 ± 14	307 ± 44	352 ± 43
Gestational age (days)	132 ± 2	131 ± 2	133 ± 2	133 ± 1	131 ± 3	133 ± 1
Sex (M/F)	6/2	3/0	3/4	3/2	2/1	4/3
Fetal plasma cortisol (µg/dL)	2.5 ± 0.6	—	2.6 ± 1.0	3.5 ± 0.8	—	2.1 ± 0.7

After ANS treatment were not different among groups.

Confocal microscopy of immunofluorescent staining of the fetal lung for the epithelial cell marker TTF-1, smooth muscle actin (SMA), and type II alveolar cell markers pro-SPC, and ABCA3 showed no differences in the relative numbers of cells positive for these markers 5 days after treatment (Supplemental Fig. 1). Further, there were no differences in the proportion of cells expressing the cell cycle marker Ki-67.

### Transcriptomic effects of the clinical drug and Beta-Ac on the fetal lung

**Figure 2:**
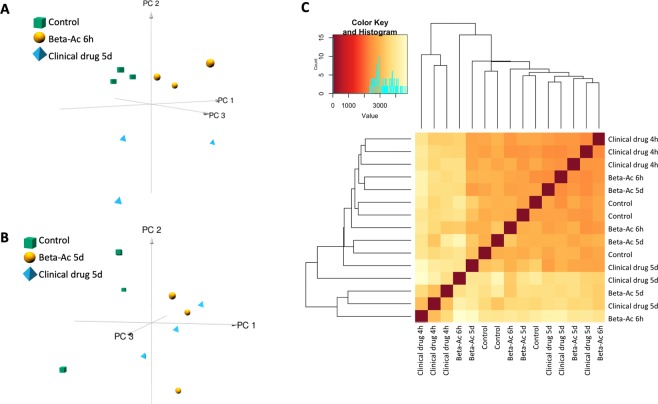
Transcriptomic analysis was performed on whole lung RNA 4 hours and 5 days after administration of the clinical drug, and 6 hours and 5 days after Beta-Ac 0.125 mg/kg and from saline-treated animals (n = 3 animals per group). 3-dimensional principal component analysis (PCA) was generated using log-transformed read counts. (**A**) At the early timepoint PCA separated the animals treated with the clinical drug at 4 h, Beta-Ac at 6 h, and controls. (**B**) At 5 days there is separation of control animals and overlap of animals treated with the clinical drug and Beta-Ac (**C**) Sample correlation heatmap displaying the similarity in gene expression profile. Red color indicates increasing sample correlation and yellow color indicates decreasing sample correlation. Dendrogram clustering indicates the overall similarity of the samples. Both analyses showed separation of the clinical drug at 4 h and overlap of animals in the other treatment groups.

RNA-sequencing analyses were performed on whole lung tissue from fetuses delivered 4 hours and 5 days after treatment with the clinical drug and 6 hours and 5 days after Beta-Ac (Fig. 2). Principal component analysis of transcriptomic changes separated animals 4 h after treatment with the clinical drug, 6 h after Beta-Ac, and control animals, with the animals treated with the clinical drug being the most distant from the others (Fig. 2A). In contrast, by 5 days the clinical drug and Beta-Ac overlapped (Fig. 2B). Further, the correlation heatmap showed the most distinct transcriptome profiles for animals treated with the clinical drug 4 h prior to delivery (Fig. 2C).

**Figure 3:**
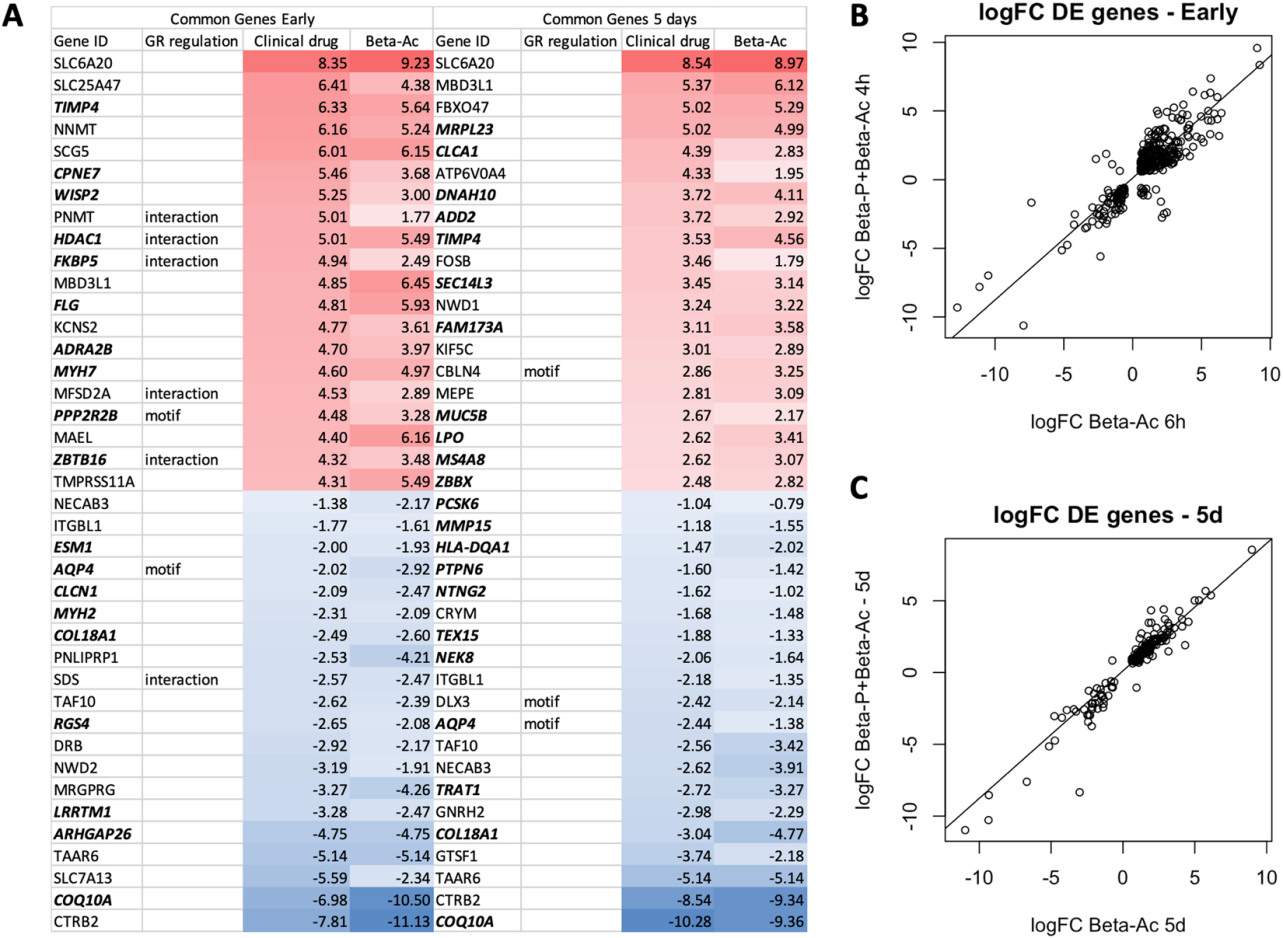
Top common differentially expressed genes in each group relative to control were determined using thresholds of p-value < 0.05, q-value < 0.1 and fold-change > 1.5. (**A**) Top 20 up and down regulated genes relative to control and predicted regulation by the glucocorticoid receptor (GR) either by the presence of a GR motif or predicted interaction by ingenuity pathway analysis. Genes reported in the literature to be associated with “lung”, “respiratory disease”, or “lung cell line” are bolded. Scatterplots of log fold-changes (logFC) of differentially expressed genes from. (**B**) Beta-Ac 6 h and the clinical drug 4 h relative to control and (**C**) Beta-Ac 5d and the clinical drug 5d relative to control. Most commonly differentially expressed genes had a similar direction and magnitude of changes. There was strong correlation between logFC values (r = 0.87 for 6 h and 4 h; r = 0.96 for 5d).

Differential expression analysis was performed using the EdgeR package on R with differentially expressed genes determined using p-value < 0.05, q-value < 0.1 and fold-change > 1.5. At the times of high fetal plasma concentration the clinical drug caused differential expression of 1,779 genes while Beta-Ac caused the differential expression of only 393 genes, of which 372 were common to both treatments. The common differentially expressed genes had similar magnitude of fold changes, with a Pearson correlation value of 0.87 (Fig. 3A,B). In contrast, after 5 days the clinical drug and Beta-Ac differentially expressed a similar number of genes (318 and 419 genes, respectively), with 185 genes differentially expressed in common. These commonly regulated genes at 5 days also had a similar magnitude of differential expression and highly correlated log fold-change values (Fig. 3A,C). By transcription factor binding site prediction and protein regulation or interaction prediction, several of the common top regulated genes at both timepoints have either a glucocorticoid receptor enhancer motif or are predicted to be regulated or interact with the NR3C1 receptor. Several common regulated genes are also reported in the literature to be associated with “lung expression”, “respiratory disease”, and “lung cell line” (Fig. 3A).

Genes differentially regulated by either of the ANS treatments were associated with several similar biological processes. At the early time point induced genes were associated with “cellular localization”, “developmental process”, “ion and protein transport” while suppressed genes were associated with “cellular morphogenesis”, “chemotaxis”, and “immune responses”.

**Figure 4:**
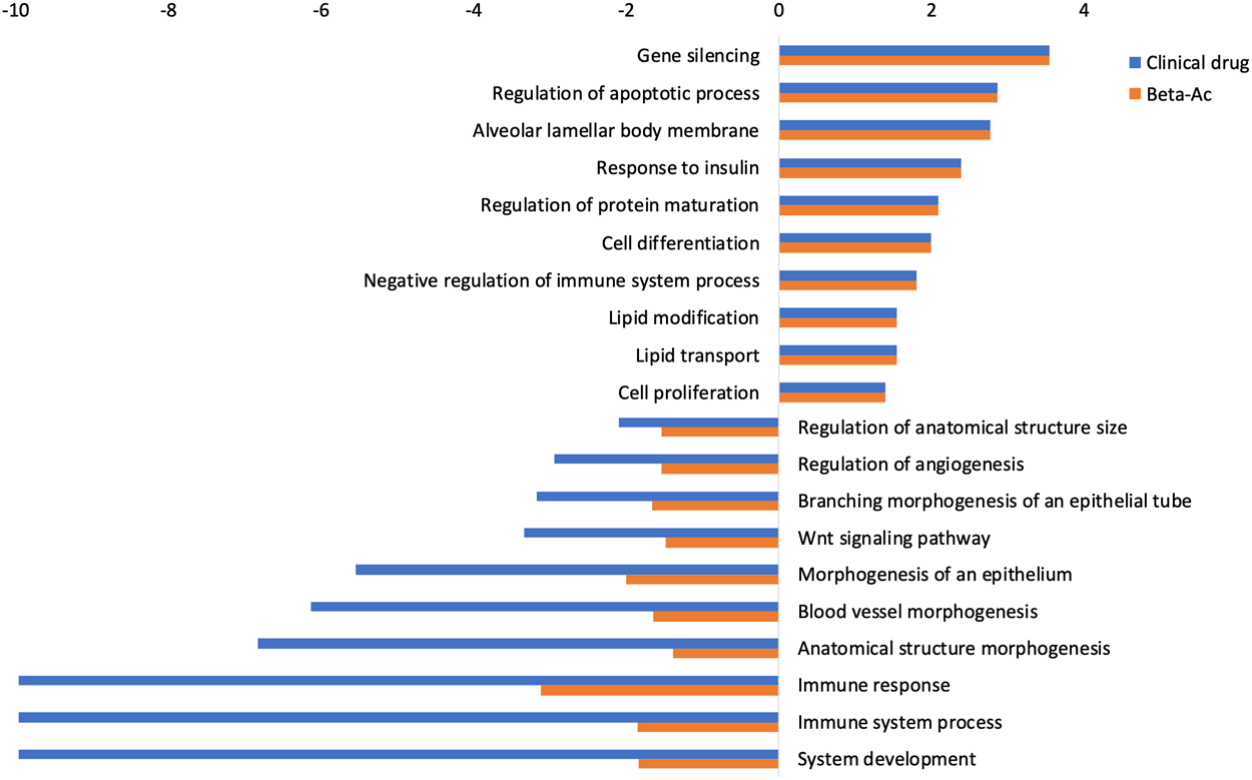
Gene set enrichment analysis comparing the clinical drug and Beta-Ac to control in the lung at time of peak Beta plasma concentration in the fetus. Selected gene ontology terms are displayed with the bar chart representing log p-values. Positive p-values denote induced genes and negative p-values denote suppressed genes. Genes suppressed by the clinical drug were more strongly associated with morphogenesis and developmental processes than genes suppressed by the Beta-Ac.

Despite the similarities, there were large differences between biological processes and pathways that were differentially regulated in the lung by the clinical drug and Beta-Ac relative to control at the time of peak drug concentration in the fetal plasma (Fig. 4). The clinical drug had larger inhibitory effects on immune system processes and development, including suppression of Th1, Th2 and Th17 lymphocyte differentiation, lymphocyte proliferation and activation, and cytokine signaling. The clinical drug also modulated biological processes related to organ development and morphogenesis, which were only weakly associated with Beta-Ac treatment. Specifically, genes inhibited by the clinical drug were strongly enriched for angiogenesis and vascular development, endothelial cell proliferation, epithelial tube morphogenesis, and canonical Wnt signaling. Genes differentially expressed by the clinical drug at the early time point may not be contributing to lung maturational responses as the 2 treatments resulted in similar improvement in lung gas volume and similar RNA profiles at 5 days.

### Transcriptomic effects in the fetal hippocampus at 5 days

**Figure 5:**
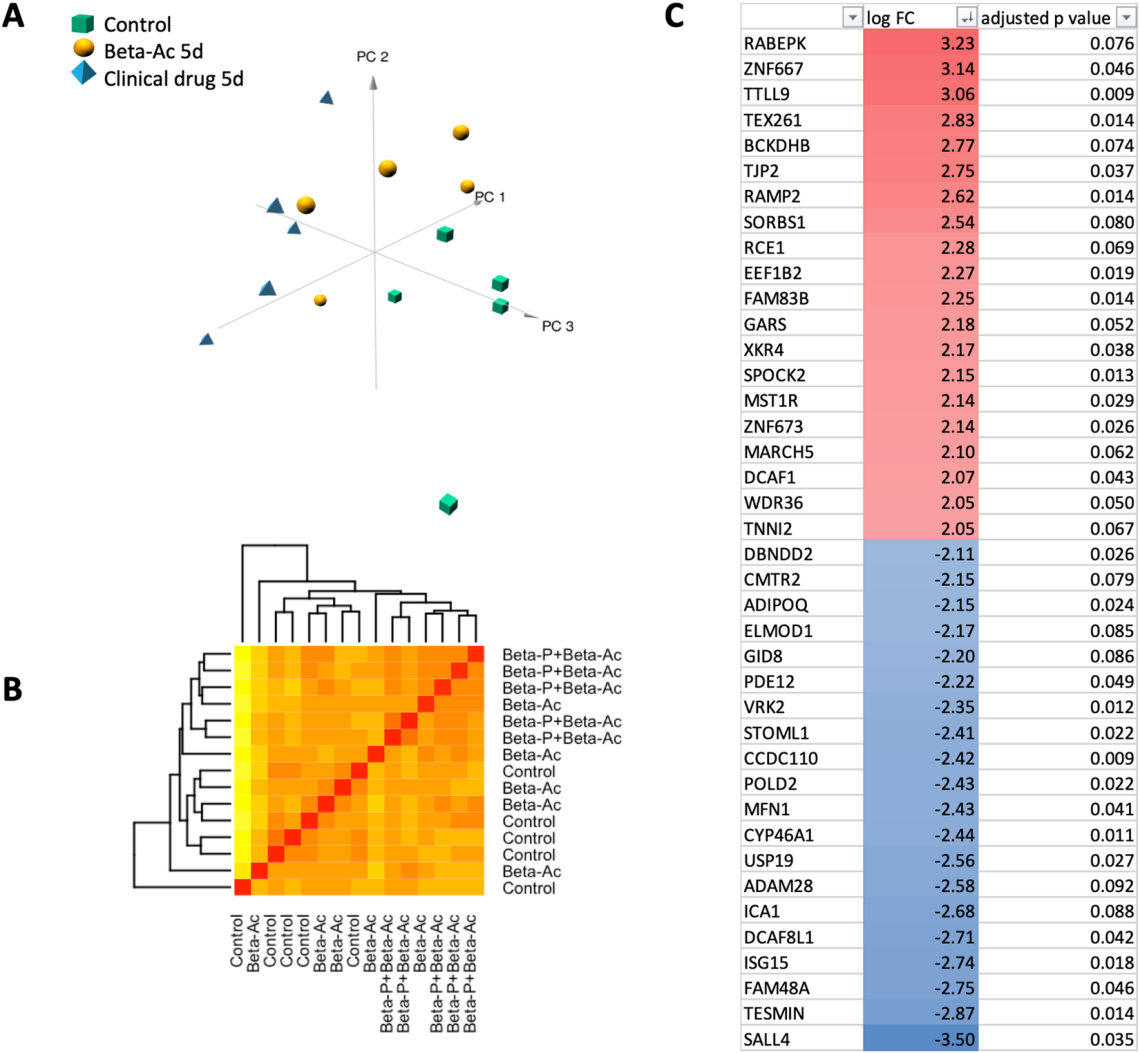
Transcriptomic analysis was performed for fetal hippocampus from saline-treated animals and 5 days after administration of ANS (n = 5 animals per group). (**A**) 3-dimensional principal component analysis (PCA) was generated using log-transformed read counts. (**B**) Sample correlation heatmap displaying the similarity in gene expression profile. Red color indicates increasing sample correlation and yellow color indicates decreasing sample correlation. Dendrogram clustering indicates the overall similarity of the samples. Both analyses showed separation of the control from animals treated with the clinical drug, while animals treated with Beta-Ac are interspersed between the controls and clinical treatment animals. (**C**) Top differentially induced and suppressed genes by the clinical drug in the hippocampus at 5 days. Genes are ordered by magnitude of gold change; p values are adjusted by the Benjamin-Hochberg method.

To evaluate potentially toxic effects of ANS on the fetal brain development we compared the transcriptome of the fetal hippocampus 5 days after ANS treatment. The principal component analysis and the correlation heatmap demonstrate that control animals cluster separately and distant from animals treated with the clinical drug (Fig. 5A,B). In contrast, animals treated with Beta-Ac 0.125 mg/kg are interspersed between the other groups but with wide variation among samples.

**Figure 6:**
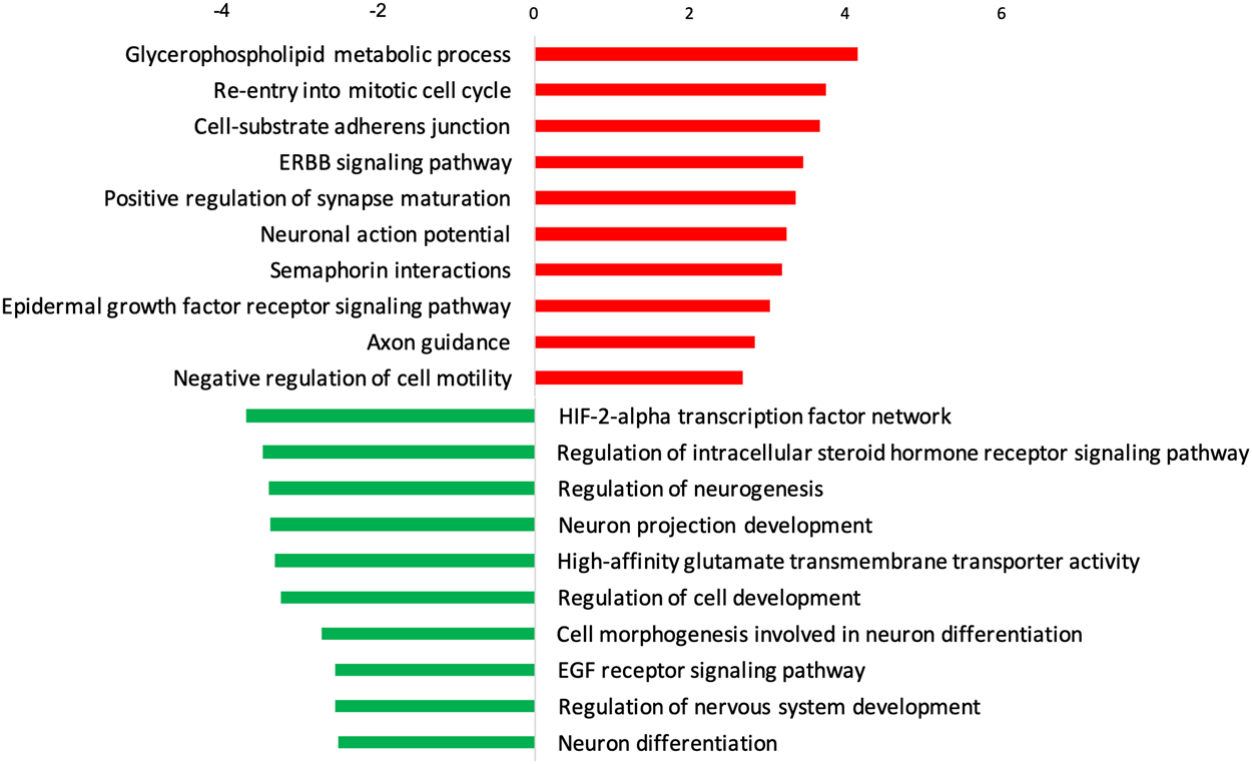
Gene set enrichment analysis comparing differentially expressed genes for the clinical drug compared to control in the fetal hippocampus. There was no differential expression between Beta-Ac and control. Selected gene ontology terms are displayed with the bar chart representing log p-values. Positive p-values denote induced genes and negative p-values denote suppressed genes.

**Figure 7:**
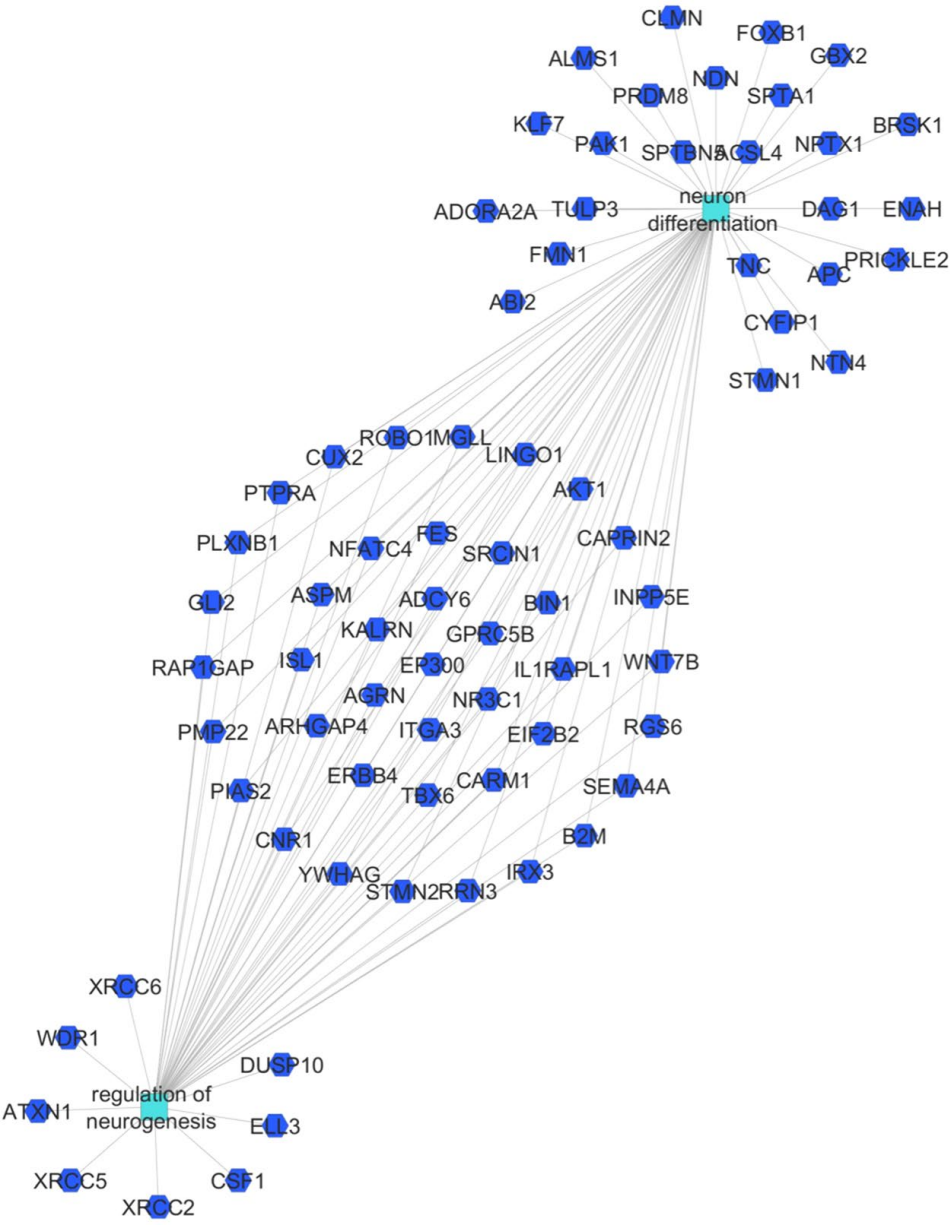
Network of genes suppressed by the clinical drug in the fetal hippocampus at 5 days associated with the biological processes of neurogenesis and neuron differentiation.

By differential expression analysis 1612 genes were differentially regulated (788 induced, 824 suppressed) in the hippocampus by the clinical drug at 5 days compared to control (Fig. 5C). There were no statistical differences between gene expression levels in the Beta-Ac treated animals compared to control. Genes induced by the clinical drug were associated with the regulation of synapse maturation and semaphorin interactions, among others. Suppressed genes were associated with regulation of neurogenesis, neuron, projection development, cellular morphogenesis and nervous system development (Figs 6 and 7).

## Discussion

Since 1972, use of ANS for women at risk of preterm labor at 24–34 weeks gestation decreased the incidence of respiratory distress syndrome and mortality in preterm newborns, but the formulation and dosing was never optimized by pharmacokinetic analyses or for safety^2,25^. In fact, the expanding use of ANS beyond the 24 to 34 week gestational has identified previously unrecognized risks such as hypoglycemia in late preterm infants and increased mortality in middle and low-resource countries^3,9^. We report that fetal lung maturation in the primate can be achieved with a single weight-based dose of Beta-Ac that avoided fetal exposure to higher Beta levels from the Beta-P component of the clinical drug. Beta-Ac minimized early transcriptional changes in the fetal lung associated with immunity and morphogenesis, and at 5 days in the fetal hippocampus associated with nervous system development despite similar pulmonary maturation. As even routine short-term use of corticosteroids is associated with risks, and complications are dose related^26^, the lowest fetal exposure sufficient to get the maturational benefit should be the goal.

Different steroid formulations and dosing intervals are used around the world based on a total dose of 24 mg with minimal experimental or clinical data to support equivalency^25^. We previously showed with fetal sheep that a single dose of Beta-Ac that was 25% of the standard 2-dose treatment with the clinical drug yielded similar improvements in gas exchange and lung compliance 2 days after treatment^23^. This treatment strategy in sheep exposes the fetus to a peak drug concentration that is 10% of the peak drug concentration with the clinical drug, while providing continuous fetal exposure to betamethasone for 24 hours^23^.

In the primate, treatments with Beta-Ac alone and the clinical drug resulted in similar mechanical induction of lung maturation as demonstrated by the pressure-volume curves and similar increase in the surfactant content, measured by the SatPC concentration in the BALF. Increased surfactant production by the premature lung decreases the severity and incidence of RDS in preterm newborns. Moreover, there were no differences between the lung transcriptomes 5 days after treatment. Interestingly, the proportions of cells expressing the epithelial marker TTF-1 or the type 2 alveolar cell markers, ABCA3 and SPC, were similar among treatment groups and control at 5 days, suggesting that ANS induced transient changes in expression of mRNA but not persistent cellular differentiation to increase the number of surfactant producing cells. This finding is consistent with the observation that the effects of ANS are transient and clinical trials have not consistently demonstrated benefit in decreasing RDS beyond 7 days after treatment^1,27^.

The early transcriptomic changes in the lung 4 to 6 hours after ACS exposure offers new insight into the complex signaling mechanism of lung maturation induced by corticosteroids. While most studies have focused on the effects of ACS on the endpoints of increased surfactant production and improved lung structure here we show that ACS has large effects on developmental, vascularization, cell signaling, and cell cycle pathways. These multiple effects likely all contribute to the improved lung function seen in preterm newborns after ACS treatment. More interestingly, 5 days after ACs treatment, transcriptional changes were limited and were associated with ion transport and cytoskeletal organization. These changes could be associated with continued structural and tissue water balance maturation. However, most of the effects of ACS on gene expression had disappeared by 5 days.

We provide new information that the high peak drug level from the clinical drug was associated with more suppression of lung immune responses, angiogenesis and developmental pathways based on transcriptome analyses. While the clinical drug regulated a larger number of genes and pathways than Beta-Ac, the genes that were commonly differentially expressed had similar magnitudes of change, indicating that the additional steroid exposure may not be contributing to more maturational signaling. Changes in expression of other genes may only have harmful harmful side effects. While in high resource environments ANS has not been associated with increased risk of maternal or neonatal infection or sepsis^28^, in the subgroup of patients with preterm rupture of membranes the risk of chorioamnionitis was increased with repeated treatments with ANS^29^. In middle and low-resource countries the increased infant mortality associated with ANS treatment may be caused by increased the risk of infection^10^. Due to cost and availability, the international cluster-randomized trial used Dex-P given as 4 intramuscular doses of 6 mg every 12 hours^9^. This dosing strategy, provides fetal exposure to ANS for greater than 48 hours but with 4 high peak fetal plasma levels that are not necessary in animal models^22,30^. Avoiding high fetal concentrations of corticosteroids should minimize the treatment effects on the maternal and fetal immune responses and the risk of perinatal infection.

More concerning are the reports of direct effects of glucocorticoids on the fetal brain. Preterm newborns exposed to glucocorticoids had decreased number of neurons in the hippocampus^12^, consistent with a previous report of increased apoptosis of neuron in the hippocampus of macaques after treatment with glucocorticoids^31^. We found that fetal exposure to the clinical drug resulted in suppression of genes associated with neurogenesis and nervous system development 5 days after treatment. There were no differences in the hippocampus transcriptome between animals treated with Beta-Ac compared to control but we did observe a wide variability in the Beta-Ac group with some animals clustering with controls and others with the clinical drug. This variability could be due to individual variations regarding drug metabolism affecting the fetal exposure to the treatment or genetic variants affecting the molecular response to corticosteroids. In our limited sample size the sex of the animal did not seem to affect the response. The most recent meta-analysis of antenatal corticosteroids showed a trend towards reduced neurodevelopmental impairment after a single course of ANS in infants less than 34 weeks gestation in high resource countries^28^. There are no data on neurodevelopmental outcomes for late preterm infants where the clinical benefits of ANS are small and may not outweigh the risks. This benefit to risk ratio may be even less for elective C-sections. Even more problematic are reports of increased renal disease, obesity and metabolic syndrome at advanced ages in sheep and baboons exposed as fetuses to ANS^32–34^. These effects cannot be evaluated in human populations being treated with ANS today and may be at long-term risk of fetal effects on adult outcomes.

Here we demonstrate that a clinically relevant dose of ANS used for fetal lung maturation caused profound and early changes in transcriptional networks that control lung development and immunity and persistent changes on brain development pathways. Many of the changes can be avoided by low-dose Beta-Ac while preserving the physiological maturational effects in a nonhuman primate model. This strategy should be considered for clinical trials to optimize ANS treatment in preterm infants and decrease potential toxic effects.

## Methods

### Animals

The Institutional Animal Care and Use Committee at the University of California Davis approved all animal procedures, which were performed at the California National Primate Research Center according to the approved protocol. Time-mated pregnant Rhesus macaques were given the clinical drug as intramuscular Celestone Soluspan® 0.25 mg/kg (6 mg/ml containing 3 mg/mL betamethasone as Beta-P and 3 mg/mL of Beta-Ac; Merck Sharp & Dohme, Kenilworth, NJ), 0.125 mg/kg Beta-Ac (a gift from Merck Sharp & Dohme, Kenilworth, NJ), 0.06 mg/kg Beta-Ac or saline prior to preterm delivery at 132 ± 2 days gestational age (term is 165 days). To investigate the early transcriptional effects, fetuses were delivered at the time of peak fetal blood Beta levels based on measurements in fetal sheep^23^: 4 h after the clinical drug and 6 h after Beta-Ac (n = 3 animals/group). Lung samples were frozen for RNA-sequencing. To assess the maturational effects of the interventions, other groups of fetuses were treated 5 days before delivery at 132 ± 2 days of gestation (n = 5–8 animals/group). After delivery, pressure-volume curves were measured with a syringe and pressure manometer by inflating the lungs to 40 cm H_2_O pressure and followed by deflation with measurements of lung volumes. The right upper lobe of the fetal lung was inflation fixed with formalin at 30 cm H_2_O pressure for histology; tissue samples from the right lower lobe of fetal lung and the hippocampus were snap frozen for RNA-sequencing

### Saturated phosphatidylcholine and cortisol measurements

Alveolar lavage fluid was recovered from the left lung and lipids were extracted with chloroform-methanol (2:1). Saturated phosphatidylcholine (SatPC) was isolated after exposure to osmium tetroxide and quantified by phosphorus assay as previously described^35^. Cord blood plasma cortisol levels were measured using an ELISA kit (EA65; Oxford Biomedical Research, Rochester Hills, MI).

### Immunofluorescence and confocal microscopy

Sections from paraffin-embedded tissues underwent heat-assisted antigen retrieval with citrate buffer (pH 6.0), followed by blocking with donkey or goat serum and incubation with primary antibodies overnight (Table 2). The following day, sections were incubated with species-specific Alexa Fluor antibody (Life Technologies, Carlsbad, CA), followed by DAPI (Life technologies, Carlsbad, CA, dilution 1:2000). Sections were mounted with ProLong Gold (Life technologies, Carlsbad, CA). Stained slides were imaged by confocal microscopy for co-localization of fluorescent antibodies at 40x magnification, 1024 × 1024 pixels resolution on a Nikon Eclipse A1RSi inverted microscope (Nikon Instruments Inc., Melville, NY). Confocal images were analyzed using Nikon NIS Elements software (Nikon Instruments Inc., Melville, NY), for object count and colocalization.

**Table 2:** Primary antibodies and dilutions for immunofluorescence of paraffin-embedded lung sections.

Antibody	Cellular marker	Species	Dilution
anti-TTF-1^a^	Epithelial	Rabbit	1:500
anti-TTF-1^a^	Epithelial	Guinea pig	1:200
anti-pro-surfactant protein C (SPC)^a^	Alveolar type II	Rabbit	1:100
anti-ABCA3^a^	Alveolar type II	Guinea pig	1:100
anti-smooth muscle actin (SMA)^b^	Smooth muscle, myofibroblast	Mouse	1:2000
Anti-Ki67^c^	Cell cycle	Rat	1:50

^a^Seven Hills Bioreagents, Cincinnati, OH; ^b^Santa Cruz Biotechnologies, Dallas, TX; ^c^LifeSpan Biosciences, Seattle, WA.

### Statistical analyses

Statistical analyses of morphological and immunofluorescence data were performed with GraphPad Prism software (Carlsbad, CA). Values for continuous variables were compared by t-test or ANOVA followed by Holm-Sidak post-hoc analysis for multiple comparisons. Data are presented as bars with individual data points and standard deviation.

### RNA isolation and sequencing

Total RNA was extracted from frozen lung tissues using the RNeasy Universal Mini Kit (Qiagen, Valencia, CA) according to the manufacturer’s instructions. RNA quality and integrity were verified using the Agilent 2100 Bioanalyzer (Agilent, Agilent Technologies, Santa Clara, CA). RNA-sequencing was performed by the Cincinnati Children’s Hospital Medical Center DNA Sequencing and Genotyping Core with a read depth of 20–30 million reads per sample for 75 bp paired-end reads. The raw sequence reads in FASTQ format were aligned to the Rhesus (Macaca mulatta) genome build MMUL1.0 using Bowtie 2^36^. Reads were counted using featureCounts^37^. After checking data quality, raw read counts were filtered to exclude genes with low expression (<7 reads) and normalized using the trimmed mean of M values method^38^. Differential expression analyses comparing treatment groups to control and between each other were performed using EdgeR^39^ followed by false discovery rate adjustment using Storey’s method^40^. Genes were considered differentially expressed based on their fold-change relative to control (= or >1.5), p-value (<0.05) and q-value (<0.1).

### Functional enrichment and pathway analysis

Differentially expressed genes were used for functional enrichment analysis of Gene Ontology and pathway terms using the ToppCluster web server^41^. Only unique terms associated with either induced or suppressed genes and at least 2 genes are reported. Negative log p-values represent terms associated with suppressed gene expression and positive log p-values are associated with induced gene expression. Promoter GRE cis-element was scanned using the Msig-DB motif gene sets within 4 kb around their transcription starting sites (http://software.broadinstitute.org/gsea/msigdb). The evidence that NR3C1 regulates or interacts with genes in the top hits list was obtained via literature mining using Genomatix co-citation database (Genomatix Inc.) and IPA knowledge base (Ingenuity Pathway Analysis, QIAGEN). Annotation of genes expressed in the lung or associated with respiratory disease were collected from IPA knowledge base.

## Supplementary information

Supplemental figure 1

## Data Availability

The gene expression data discussed in this publication have been deposited in NCBI’s Gene Expression Omnibus and are accessible through GEO Series Accession Number GSE118438 (https://www.ncbi.nlm.nih.gov/geo/query/acc.cgi?acc=GSE118438).
