# Supplementary material for: Dosing and formulation of antenatal corticosteroids for fetal lung maturation and gene expression in rhesus macaques
Source: Sci Rep. 2019 Jun 21;9:9039. doi: 10.1038/s41598-019-45171-6 (PMC6588577; doi:10.1038/s41598-019-45171-6)

**Dosing and formulation of antenatal corticosteroids for fetal lung maturation and gene expression in rhesus macaques**

Augusto F SCHMIDT*^1,2^, Paranthaman S KANNAN ^1^, James P BRIDGES ^1,2^, Alyssa FILUTA ^1^, Dakota LIPPS ^1,2^, Matthew KEMP ^3^, Lisa A. MILLER ^4^, Suhas G KALLAPUR ^5^, Yan XU ^1,2^, Jeffrey A WHITSETT ^1,2^, Alan H JOBE ^1,2^

1. Division of Neonatology, Perinatal and Pulmonary Biology, Cincinnati Children’s Hospital Medical Center, OH
2. Department of Pediatrics, College of Medicine, University of Cincinnati, OH
3. School of Women’s and Infants’ Health, University of Western Australia, Perth, Australia
4. Department of Anatomy, Physiology & Cell Biology, School of Veterinary Medicine, University of California, Davis, CA
5. Division of Neonatology and Developmental Biology, David Geffen School of Medicine, University of California, Los Angeles, CA

**Supplemental figure 1.** Representative photomicrographs of sections from rhesus lungs stained for immunofluorescence and confocal microscopy analysis (top panel). The total number of cells and cells positive for **A**: TTF-1, **B**: pro-SPC, **C**: ABCA3, **D**: Ki67 and **E**: Smooth muscle actin (SMA) were counted with automated system and were not different. There was no difference in the fractional area stained for SMA (**F**).
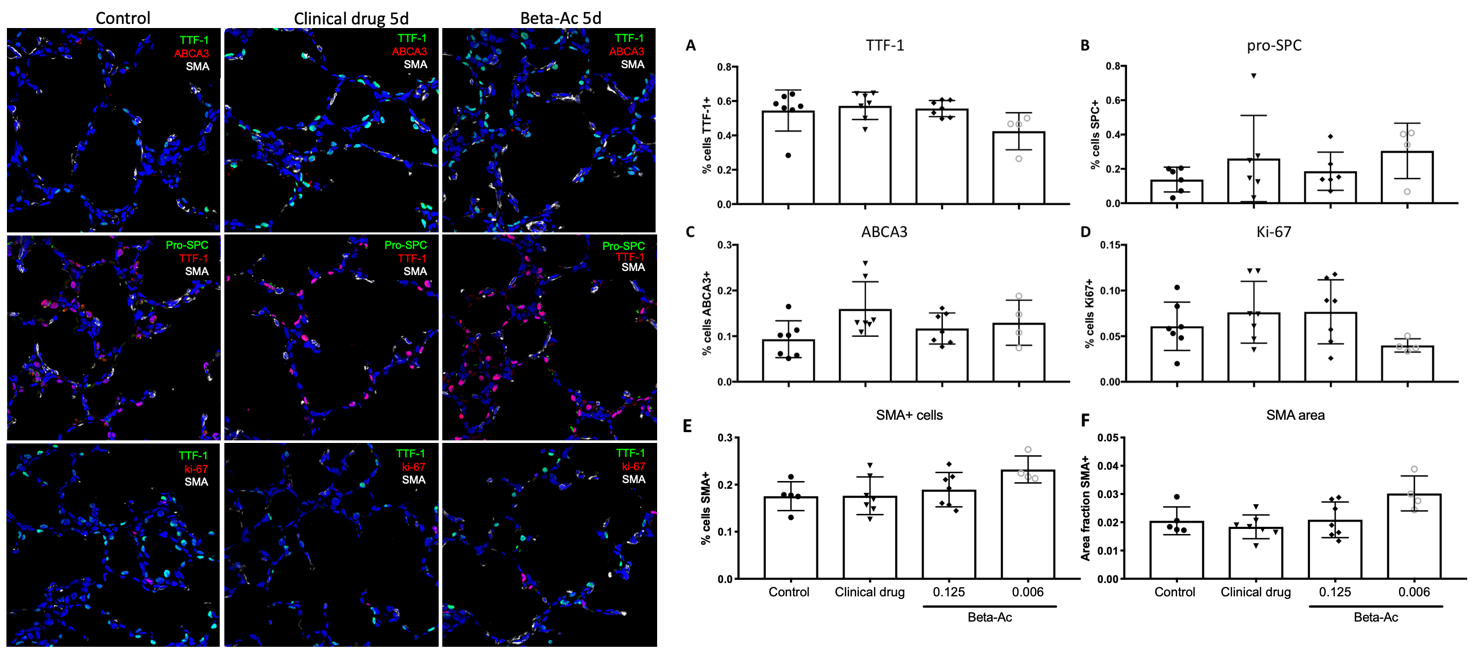

Supplement: Supplementary file 1 — Supplemental figure 1 [file 41598_2019_45171_MOESM1_ESM.docx]
